# Malnutrition- inflammation- atherosclerosis (MIA) syndrome associates with periodontitis in end-stage renal disease patients undergoing hemodialysis: a cross-sectional study

**DOI:** 10.1038/s41598-023-38959-0

**Published:** 2023-07-21

**Authors:** Risako Mikami, Koji Mizutani, Tomohito Gohda, Yusuke Matsuyama, Hiromichi Gotoh, Keita Nakagawa, Shu Takemura, Norio Aoyama, Takanori Matsuura, Daisuke Kido, Kohei Takeda, Natsumi Saito, Yuichi Izumi, Takanori Iwata

**Affiliations:** 1 Department of Periodontology, Graduate School of Medical and Dental Sciences, Tokyo Medical and Dental University (TMDU), 1-5-45 Yushima, Bunkyo-Ku, Tokyo, 113-8549 Japan; 2 Department of Nephrology, Juntendo University Faculty of Medicine, Tokyo, Japan; 3 Department of Oral Health Promotion, Graduate School of Medical and Dental Sciences, Tokyo Medical and Dental University (TMDU), Tokyo, Japan; 4 Department of Internal Medicine, Saiyu Soka Hospital, Saitama, Japan; 5 Department of Periodontology, Kanagawa Dental University, Kanagawa, Japan; 6 Weintraub Center for Reconstructive Biotechnology, Division of Regenerative and Reconstructive Sciences, UCLA School of Dentistry, Los Angeles, CA USA; 7 Oral Diagnosis and General Dentistry, Tokyo Medical and Dental University Hospital, Tokyo, Japan; 8 Oral Care Periodontics Center, Southern TOHOKU General Hospital, Fukushima, Japan

**Keywords:** Periodontitis, Occlusion, Oral conditions, Periodontics, Haemodialysis

## Abstract

Malnutrition-inflammation-atherosclerosis (MIA) syndrome is a significant risk factor for mortality in patients undergoing hemodialysis. This study aimed to investigate the association between MIA syndrome and oral health status in hemodialysis patients. A cross-sectional study was conducted on 254 hemodialysis patients. Comprehensive medical and dental examinations were performed. Three components were included to define MIA syndrome: Geriatric Nutritional Risk Index, serum high-sensitivity C-reactive protein, and history of cardiovascular events as indicators of malnutrition, inflammation, and atherosclerosis, respectively. The association of MIA syndrome components with periodontitis and occlusal support was examined by multiple-ordered logistic regression analysis. Of 254 participants, 188 (74.0%) had at least one component of MIA syndrome. After adjusting for possible confounding factors, severe periodontitis was significantly associated with presence of more components of MIA syndrome (odds ratio [OR]: 2.64, 95% confidence interval [CI], 1.44–4.84, p = 0.002) and inflammation and malnutrition components (OR: 2.47 and 3.46, 95% CI 1.16–5.28 and 1.70–7.05, p = 0.020 and 0.001). On the other hand, occlusal support, evaluated by Eichner index, was not significantly associated with MIA syndrome or any of its components. In conclusion, periodontitis is associated with MIA syndrome, particularly with inflammation and malnutrition in hemodialysis patients, independent of occlusal support.

## Introduction

End-stage renal disease (ESRD) patients undergoing hemodialysis have reported a high annual crude mortality rate of over 20% due to infections, degradation of immune systems^1^, and cardiovascular diseases (CVDs)^1,2^. These fatal compound situations comprise the Malnutrition-inflammation-atherosclerosis (MIA) syndrome, which has a significant impact on the survival prognosis of hemodialysis patients^3^. Generally, hemodialysis patients have systemic chronic inflammation, which is the main feature of MIA syndrome. Another critical factor of MIA syndrome is malnutritional state. Protein energy wasting (PEW) is a nutritional disorder characterized by decreased body protein and energetics^4^, and 28–54% of dialysis patients have PEW^5^. Atherosclerotic disease develops at a high rate in patients with PEW and chronic inflammation; this pathological situation is defined as MIA syndrome^6^. Malnutrition, inflammation, and atherosclerosis worsen progressively and independently. Simultaneously, they interact to form a vicious cycle that accelerates the pathogenesis of MIA syndrome. However, no reliable treatment for MIA syndrome has been established so far.

Periodontitis is a major cause of tooth loss in adults. Tooth loss directly decreases occlusal contacts, leading to poor nutritional status^7^ owing to impaired chewing ability^8^ and unbalanced food selection^9^. As ESRD patients undergoing hemodialysis have poor oral health and fewer remaining teeth^10^, it is inferred that their nutrient intake is less efficient than that of healthy individuals. Furthermore, periodontitis increases systemic inflammation^11^ and is a risk factor for atherosclerosis^12^. Although the detailed mechanism remains unclear, periodontitis has been shown to affect the progression of CVD^13^ and be a risk factor for atherosclerosis^12^. According to the systematic review, periodontal therapy has shown improvement in atherosclerotic profiles, including serum levels of inflammatory cytokines and lipid metabolism markers^14^.

Previously, we have demonstrated a significant association of periodontopathogenic bacteria, *Porphyromonas gingivalis* (*P. gingivalis*) infection with circulating tumor necrosis factor receptors (TNFRs), which is an inflammatory marker and predictor of mortality in hemodialysis patients^15,16^. In addition, a longitudinal study revealed that poor oral hygiene significantly affects the mortality of hemodialysis patients^17^. The previous studies demonstrated the association between periodontitis and inflammatory and nutritional status^18^ and death from cardiovascular causes^19^. The background of these clinical findings suggests that poor oral health may be involved in MIA syndrome^20^. Occlusal support can be deteriorated among patients with severe periodontitis and influences on nutritional status. To the best of our knowledge, there are no studies that examine the impact of periodontal disease combined with occlusal support on the inflammatory and nutritional status of patients undergoing hemodialysis. We hypothesize that periodontitis and loss of occlusal contacts can be factors aggravating MIA syndrome in ESRD patients undergoing hemodialysis. The aim of this study is to evaluate the association between MIA syndrome and oral condition, particularly periodontitis and occlusal status in ESRD patients undergoing hemodialysis, which, to the best of our knowledge, has not been examined in any study.

## Materials and methods

### Study participants

A cross-sectional study was conducted at an outpatient hemodialysis clinic located in Saitama prefecture, Japan in April 2015. Overall, 266 ESRD patients undergoing maintenance hemodialysis for at least three month who provided written informed consent were included in this study. This study was approved by the Research Ethics Committee of Tokyo Medical and Dental University (D2014-126) and was conducted in accordance with the Declaration of Helsinki (revised in 2013). Information on medical history, body mass index (BMI), blood pressure, and biochemical tests including high sensitivity C-reactive protein (hsCRP) were recorded. The dental examinations were performed in 254 participants excluding 11 patients who lacked blood biochemical test report and one patient with acute inflammation. Acute inflammation was defined as an hsCRP ≥ 10 mg/dL^21^.

### Dental examination

Experienced periodontists examined periodontal probing pocket depth, clinical attachment level, and bleeding on probing at six sites on all remaining teeth. Periodontal disease status was classified according to the American Academy of Periodontology Classification at the exmination^22^ (Supplementary Table 1). A binary variable indicating the presence of severe periodontitis was used as an explanatory variable in the analysis.

If corresponding teeth were retained in both maxilla and mandible, they were counted as an occlusal pair. The distribution of occlusal contacts was classified using the Eichner classification^23^. The Eichner index is based on the presence or absence of occlusal contact in each of the premolar and molar regions, which are called supporting zones. A maximum of four supporting zones can be present, each of which must have at least one tooth in contact with a corresponding tooth in order to be counted. In this study, the participants were divided into three groups according to the Eichner index as follows: A (four supporting zones), B (one to three supporting zones, or anterior tooth contact but no supporting zones), and C (no occlusal contact among the few remaining teeth). This variable was used as an explanatory variable in the analysis.

### Definition of MIA components

MIA syndrome is a complex involving malnutrition, inflammation, and atherosclerosis; although, no diagnostic criteria have been established yet. Therefore, we defined each of the three components, malnutrition, inflammation, and atherosclerosis, and calculated the total number of components for each participant according to previous studies^3,24,25^. Each component was defined as follows: participants who exhibited low Geriatric Nutritional Risk Index (GNRI) (< 92) were categorized into the malnutrition group^26^; participants who had high serum C-reactive protein (CRP) levels (> 0.3 mg/dL) were categorized into the inflammation group^27^; participants who underwent invasive procedures (percutaneous coronary interventions [PCI], coronary artery bypass-grafting [CABG], or percutaneous transluminal angioplasty [PTA]) for atherosclerotic diseases or had history of cardiovascular events (acute myocardial infarction [AMI], cerebral infarction, and cerebral hemorrhage) were categorized into the atherosclerosis group. GNRI is calculated with the following formula: 14.89 × serum albumin (g/dL) + 41.7 × body weight (kg)/ideal body weight (IDW) (kg), which is an assessment tool for evaluating older patients^28^. The IDW is calculated as follows: height (cm) − 100 − ((height (cm) − 150)/4 (for male) or 2.5 (for female)). From these GNRI values, 4 grade of nutrition-related risk was defined: major risk (GNRI: < 82), moderate risk (GNRI: 82–92), low risk (GNRI: 92–98), and no risk (GNRI: > 98). The number of MIA component(s) was calculated as follows; when no components (malnutrition, inflammation, and atherosclerosis) were applicable, it was set to 0, when any components were applicable, the number of applicable components were set as the number of MIA component(s) (range: 1–3). The number(s) of components for MIA syndrome and binary variable indicating each component were applied as the outcome variable in this study. We conducted a sensitivity analysis for the outcome categorized with serum albumin levels as malnutrition group. In this the analysis, participants with low serum albumins (< 3.5 g/dL) were categorized into the malnutrition group for sensitivity analysis. The total number of components for MIA syndrome was calculated as in the main analysis.

### Statistical analysis

Continuous variables are summarized as mean values and standard deviations (SD) or median and quartile values; categorical variables are summarized as numbers and percentages. ANOVA test, Kruskal–Wallis test, or Fisher’s exact tests were used for comparison between groups classified according to severity of periodontitis. Multiple ordered logistic regression analysis was performed to examine the association of severe periodontitis and Eichner index with the number of components of MIA syndrome that were present. Brant test was performed to confirm that the proportional odds assumption was not violated^29^. Multiple binary logistic regression analysis was performed to assess the association between severe periodontitis and each component of MIA syndrome. The multivariate analysis was adjusted for age, sex, BMI, smoking status, diabetes, hemodialysis vintage, and serum hemoglobin level. A p-value < 0.05 was considered reflective of statistical significance. Statistical analyses were performed using STATA software (STATA software, version 16.0, Stata College Station, TX).

### Ethical approval

All procedures performed in studies involving human participants were in accordance with Research Ethics Committee of Tokyo Medical and Dental University (D2014-126).

## Results

### Clinical characteristics of study patients

The characteristics of 254 participants are shown in Table 1. The mean age of the participants was 67.4 (SD: 12.1) years old, 167 (65.8%) were men. The causative diseases of hemodialysis were diabetic kidney disease (n = 120, 47.2%), nephrosclerosis (n = 56, 22.0%), chronic glomerulonephritis (n = 52, 20.5%), polycystic kidney disease (n = 7, 2.8%), and others (n = 19, 7.5%). The mean hemodialysis vintage was 6.7 (SD: 6.0) years.

**Table 1:** Characteristics of participants.

Characteristics		All (N = 254)	Periodontally healthy (n = 53)	Mild periodontitis (n = 71)	Moderate periodontitis (n = 83)	Severe periodontitis (n = 47)	p-value*
		Mean (SD), Median (25, 75%), or n (%)	Mean (SD), Median (25, 75%), or n (%)	Mean (SD), Median (25, 75%), or n (%)	Mean (SD), Median (25, 75%), or n (%)	Mean (SD), Median (25, 75%), or n (%)	
Male		167 (65.8%)	35 (66.0%)	41 (57.8%)	59 (71.1%)	32 (68.1%)	0.36
Age (years)		67.4 (12.1)	70.8 (11.6)	65.4 (13.2)	66.0 (12.7)	68.5 (8.7)	0.06
Smoker	Never and former smoker	212 (83.5%)	42 (79.3%)	60 (84.5%)	70 (85.1%)	40 (85.1%)	0.84
	Current smoker	42 (16.5%)	11 (20.8%)	11 (15.5%)	13 (15.7%)	7 (14.9%)	
BMI (kg/m^2^)	< 18.5	44 (17.3%)	7 (13.2%)	17 (23.9%)	8 (9.6%)	12 (25.5%)	0.04
	≥ 18.5	210 (82.7%)	46 (86.8%)	54 (76.1%)	75 (90.4%)	35 (74.5%)	
Original diseases	DKD	120 (47.2%)	34 (64.2%)	29 (40.6%)	33 (39.8%)	24 (51.1%)	0.43
	Nephrosclerosis	56 (22.0%)	9 (17.0%)	16 (22.5%)	22 (26.5%)	9 (19.1%)	
	CGN	52 (20.5%)	6 (11.3%)	16 (22.5%)	20 (24.1%)	10 (21.3%)	
	PKD	7 (2.8%)	1 (1.9%)	2 (2.8%)	2 (2.4%)	2 (4.2%)	
	Other	19 (7.5%)	3 (5.7%)	8 (11.3%)	6 (7.2%)	2 (4.2%)	
HD vintage (yrs)		5.3 (2.4, 8.9)	4.3 (2.4, 7.0)	5.8 (2.9, 8.7)	5.4 (2.1, 11.2)	5.3 (2.7, 9.8)	0.25
Diabetes		133 (52.4%)	36 (67.9%)	33 (46.5%)	36 (43.4%)	28 (59.6%)	0.02
Prior CVD		62 (24.4%)	11 (20.8%)	21 (29.6%)	18 (21.7%)	12 (25.5%)	0.62
SBP (mmHg)		148.9 (24.8)	147.4 (22.1)	145.6 (24.5)	153.3 (24.9)	147.7 (27.8)	0.24
DBP (mmHg)		78.2 (15.3)	77.3 (15.9)	78.9 (16.0)	80.1 (14.1)	74.8 (15.7)	0.28
WBC (10^3^/μL)		6.2 (2.1)	6.2 (2.0)	6.0 (2.3)	6.1 (1.9)	6.9 (2.1)	0.13
Hemoglobin (g/dL)		11.0 (2.0)	11.0 (0.9)	10.5 (1.5)	11.3 (2.8)	11.1 (1.3)	0.07
Albumin (g/dL)		3.5 (0.4)	3.4 (0.3)	3.4 (0.4)	3.5 (0.3)	3.4 ± 0.4	0.12
UA (mg/dL)		6.7 (1.4)	7.1 (1.2)	6.6 (1.4)	6.8 (1.5)	6.5 (1.4)	0.15
hsCRP (mg/dL)		0.15 (0.05, 0.43)	0.14 (0.05, 0.32)	0.15 (0.04, 0.40)	0.14 (0.04, 0.35)	0.34 (0.09, 0.81)	0.01
GNRI		90.4 (6.6)	90.4 (6.1)	89.6 (7.7)	91.8 (5.2)	89.0 (7.1)	0.08
Dental health status							
Number of teeth		19 (7, 25)	0 (0, 1)	21 (11, 26)	22 (18, 26)	17 (14, 23)	< 0.001
Number of occlusal pairs		7 (0, 11)	0 (0, 0)	9 (2, 12)	9 (5, 12)	6 (1, 10)	< 0.001
Eichner classification	A	74 (29.1%)	4 (7.6%)	27 (38.0%)	33 (39.8%)	10 (21.3%%)	< 0.001
	B	109 (42.9%)	6 (11.3%)	31 (43.7%)	42 (50.6%)	30 (63.8%)	
	C	71 (28.0%)	43 (81.1%)	13 (18.3%)	8 (9.6%)	7 (14.9%)	
Edentulous		39 (15.4%)	39 (72.2%)	0 (0.0%)	0 (0.0%)	0 (0.0%)	< 0.001
PPD ≥ 7 mm (%)**		0 (0, 0)	0 (0, 0)	0 (0, 0)	0 (0, 0)	0 (0, 1.8)	< 0.001
PPD 4–6 mm (%)**		2.9 (0.5, 9.4)	0 (0, 0)	0 (0, 1.1)	4.3 (2.4 8.0)	16.7 (9.8, 35.1)	< 0.001
PPD ≤ 3 mm (%)**		97.0 (90.6, 99.5)	100.0 (100.0, 100.0)	100 (98.9, 100.0)	95.7 (92.0, 97.6)	80.2 (62.1, 90.2)	< 0.001
BOP (%)**		7.6 (3.1, 19.0)	3.1 (0.7, 4.3)	4.3 (1.9, 11.8)	8.8 (4.3, 20.4)	15.9 (8.0, 39.5)	< 0.001

*SD* standard deviation, *BMI* body mass index, *DKD* diabetic kidney disease, *CGN* chronic glomerulonephritis, *PKD* polycystic kidney disease, *HD* hemodialysis, *CVD* cardiovascular disease, *SBP* systolic blood pressure, *DBP* diastolic blood pressure, *WBC* white blood cells, *Hb* hemoglobin, *UA* uric acid, *hsCRP* high sensitivity c-reactive protein, *PPD* probing pocket depth, *BOP* bleeding on probing.

*ANOVA, Kruskal–Wallis test, or Fisher's exact test.

**n = 215 (excluding edentulous individuals).

Among 254 participants, 53 (20.9%), 71 (28.0%), 83 (32.7%), and 47 (18.5%) participants were identified as periodontally healthy, mild, moderate, and severe periodontitis cases, respectively. In the periodontally healthy cases, 39 edentulous participants were included. The number of remaining teeth of the participants was 19 (median, quartiles: 7, 25), and the number of occlusal pairs was 7 (median, quartiles: 0, 11). According to the Eichner classification, 70.9% of the participants were classified as class B or C, and only 29.1% of the participants had all molar occlusal supporting zone.

**Figure 1:**
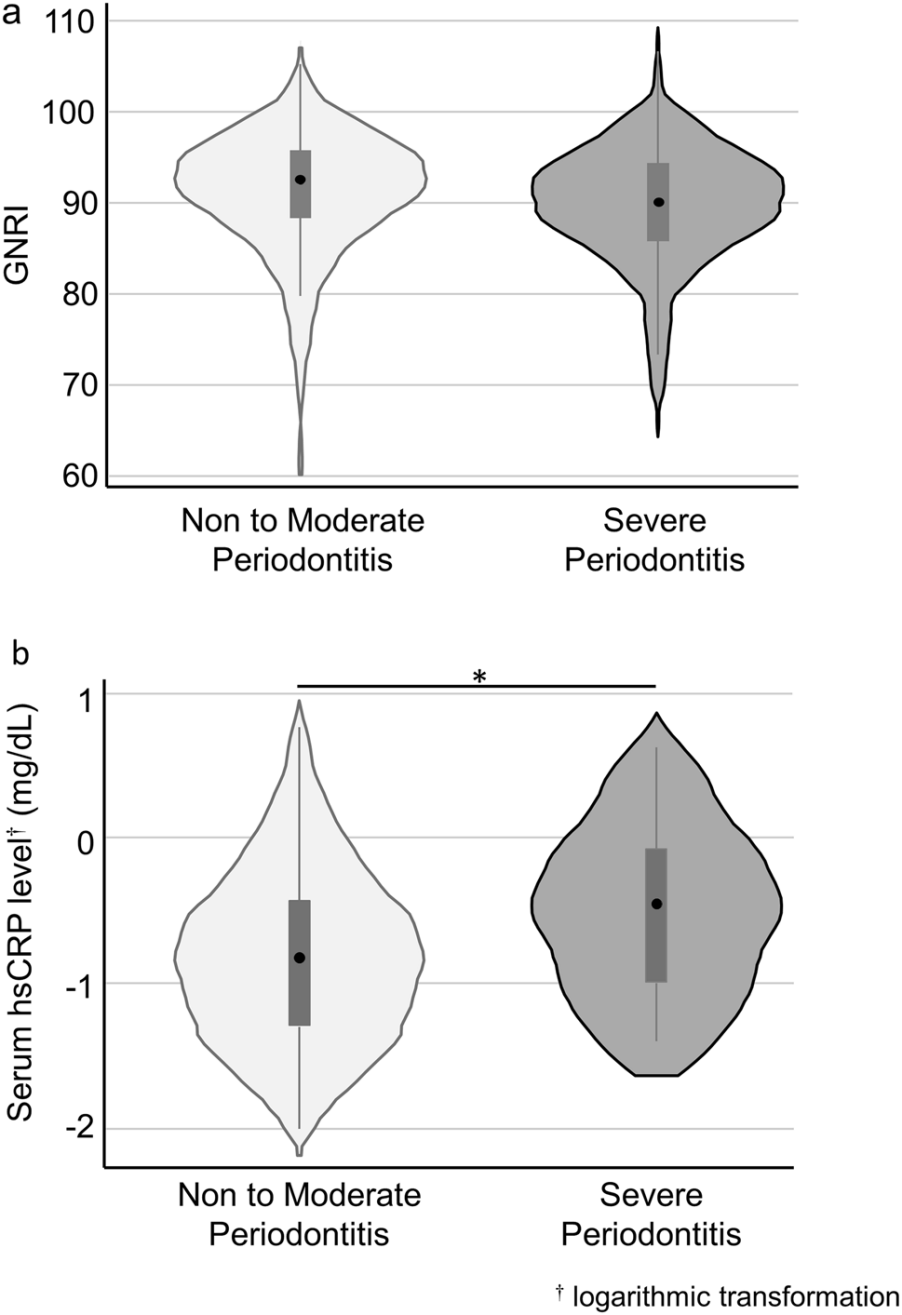
Violin/box plots illustrating Geriatric Nutritional Risk Index (GNRI) and hsCRP levels by the severity of periodontitis. (**a**) GNRI according to the severity of periodontitis (p = 0.073), (**b**) Serum hsCRP level according to the severity of periodontitis (p = 0.001). For the box plot, the top, middle and bottom lines of the box show 75 percentile, median, and 25 percentile, respectively. The whiskers indicate upper/lower adjacent values. *p < 0.05 (Mann–Whitney U test).

The mean serum GNRI were 90.4 (SD: 6.6). The GNRI of the participants with severe periodontitis tended to be lower than that of those with healthy to moderate periodontitis (Fig. 1a, p = 0.073). The median of serum hsCRP levels was 0.15 (quartiles: 0.05, 0.43) mg/dL. Compared to the healthy to moderate periodontitis cases, the severe periodontitis cases showed higher hsCRP levels (Fig. 1b, p = 0.001). In terms of the history of CVDs, 29 (11.4%), 7 (2.8%), 10 (3.9%), 15 (5.9%), 23 (9.1%), and 9 (4.3%) participants had a history of PCI, CABG, PTA, AMI, cerebral infarction, or cerebral hemorrhage, respectively.

### Association between MIA syndrome and severe periodontitis

Of the 254 participants, malnutrition, inflammation, and atherosclerosis were observed in 136 (53.5%), 81 (31.9%), and 62 (24.4%) participants, respectively. The participants with 0, 1, 2, and 3 components of MIA syndrome were 66 (26.0%), 117 (46.1%), 51 (20.1%), and 20 (7.9%), respectively. Regarding the association with periodontitis, malnutrition, and inflammation, malnutrition had tendency to be more prevalent in the participants with severe periodontitis, and inflammation was significantly more prevalent in the participants with severe periodontitis (Table 2). The number of MIA components tended to be more distributed in participants with severe periodontitis (Table 2). Multiple ordered logistic regression analysis showed that the participants with severe periodontitis had significantly higher odds ratio (OR) for an increase in the number of MIA components even after adjusting occlusal status, age, sex, smoking status, BMI, diabetes, and hemodialysis vintage (Table 3, multivariate model, OR: 2.64, 95% confidence interval [CI], 1.44–4.84, p = 0.002). In contrast, occlusal supports, as expressed by Eichner index, were shown not to be significantly associated with an increase in the number of MIA components, though the association was significant in a univariate analysis (Table 3). Age (OR: 1.03, 95% CI 1.01–1.06, p = 0.004), low BMI (OR: 3.81, 95%CI 1.88–7.73, p < 0.001), and diabetes (OR: 1.72, 95% CI 1.03–2.86, p = 0.037) were found to be significantly associated with an increase in the number of MIA components.

**Table 2:** MIA components and severe periodontitis.

	All (N = 254)	Periodontally healthy (n = 53)	Mild periodontitis (n = 71)	Moderate periodontitis (n = 83)	Severe periodontitis (n = 47)	p-value*
	n (%)	n (%)	n (%)	n (%)	n (%)	
Malnutrition	136 (53.5%)	29 (54.7%)	39 (54.9%)	36 (43.4%)	32 (68.1%)	0.057
Inflammation	81 (31.9%)	14 (26.4%)	20 (28.3%)	22 (26.5%)	25 (53.2%)	0.007
Atherosclerosis	62 (24.4%)	11 (20.8%)	21 (29.6%)	18 (21.7%)	12 (25.5%)	0.62
Numbers of MIA component(s)						
0	66 (26.0%)	16 (30.2%)	20 (28.2%)	26 (31.3%)	4 (8.5%)	0.082
1	117 (46.1%)	23 (43.4%)	30 (42.3%)	41 (49.4%)	23 (48.9%)	
2	51 (20.1%)	11 (20.8%)	13 (18.3%)	13 (15.7%)	14 (29.8%)	
3	20 (7.9%)	3 (5.7%)	8 (11.3%)	3 (3.6%)	6 (12.8%)	

Number of MIA component(s) was defined as the number of relevant components among three components of MIA syndrome (malnutrition, inflammation, and atherosclerosis).

Malnutrition: Geriatric Nutritional Risk Index < 92.

Inflammation: high sensitivity C-reactive protein > 0.3 mg/dL.

Atherosclerosis: underwent invasive procedures for atherosclerotic diseases or had history of cardiovascular events.

*Fisher's exact test.

**Table 3:** Ordered logistic regression analysis of the factors influencing the number of MIA component(s) (N = 254).

		Univariate model			Multivariate model*		
		OR	95% CI	p-value	OR	95% CI	p-value
Periodontitis	Healthy to moderate	Ref			Ref		
	Severe	2.63	1.47, 4.70	0.001	2.64	1.44, 4.84	0.002
Eichner index	A	Ref			Ref		
	B	1.43	0.82, 2.48	0.21	0.91	0.50, 1.63	0.74
	C	2.08	1.13, 3.82	0.018	1.31	0.64, 2.66	0.46
Age (years)		1.04	1.02, 1.06	< 0.001	1.03	1.01, 1.06	0.004
Female		0.99	0.61, 1.59	0.97	0.99	0.59, 1.63	0.95
Smoking Status	Never and former	Ref			Ref		
	Current	0.99	0.62, 1.59	0.97	0.83	0.43, 1.58	0.56
BMI	≥ 18.5	Ref			Ref		
	< 18.5	3.05	1.56, 5.99	0.001	3.81	1.88, 7.73	< 0.001
Diabetes		1.58	1.00, 2.50	0.051	1.72	1.03, 2.86	0.037
Hemodialysis vintage (years)		1.00	0.96, 1.04	0.95	1.00	0.97. 1.05	0.76
Hemoglobin (g/dL)		0.97	0.87, 1.09	0.64	0.98	0.87, 1.09	0.69

OR, odds ratio; CI, confidence interval; BMI, body mass index; HD, hemodialysis.

In this model, the number of MIA component(s) (0, 1, 2, and 3) is set as the ordinal dependent variable.

*Adjusted for all listed variables.

Furthermore, the association between each component of the MIA syndrome and severe periodontitis or occlusal support was evaluated (Table 4). It was shown that severe periodontitis was significantly associated with malnutrition and inflammation, even after adjustment for confounders (Table 4a and b, multivariate model, OR: 2.47 and 3.46, 95% CI 1.16–5.28 and 1.70–7.05, p = 0.020 and 0.001). However, occlusal support was found not to be significantly associated with all three components with adjustment of confounders.

**Table 4:** The association between oral health status and 3 components of MIA syndrome (N = 254).

		Univariate model			Multivariate model*		
		OR	95% CI	p-value	OR	95% CI	p-value
(a) Malnutrition							
Periodontitis	Healthy to moderate	Ref			Ref		
	Severe	2.11	1.08, 4.13	0.029	2.47	1.16, 5.28	0.020
Eichner index	A	Ref			Ref		
	B	1.18	0.65, 2.13	0.59	0.75	0.36, 1.56	0.45
	C	1.93	0.99, 3.74	0.053	1.20	0.51, 2.82	0.68
(b) Inflammation							
Periodontitis	Healthy to moderate	Ref			Ref		
	Severe	3.06	1.60, 5.87	0.001	3.46	1.70, 7.05	0.001
Eichner index	A	Ref			Ref		
	B	1.01	0.53, 1.90	0.99	0.73	0.35, 1.53	0.41
	C	1.13	0.56, 2.27	0.73	1.17	0.50, 2.73	0.72
(c) Atherosclerosis							
Periodontitis	Healthy to moderate	Ref			Ref		
	Severe	1.08	0.52, 2.23	0.84	0.78	0.35, 1.74	0.551
Eichner index	A	Ref			Ref		
	B	2.66	1.21, 5.82	0.014	1.73	0.74, 4.04	0.20
	C	2.51	1.08, 5.83	0.033	1.09	0.41, 2.89	0.87

*OR* odds ratio, *CI* confidence interval.

Malnutrition: Geriatric Nutritional Risk Index < 92.

Inflammation: high sensitivity  C-reactive protein > 0.3 mg/dL.

Atherosclerosis: underwent invasive procedures for atherosclerotic diseases or had history of cardiovascular events.

*Adjusted for age, sex, smoking status, BMI, diabetes, hemodialysis vintage, hemoglobin.

Similar results were found in the sensitivity analyses conducted using serum albumin levels to define malnutrition. Malnutrition was observed in 121 (47.6%) in 254 participants. Multiple ordered logistic regression analysis showed that the participants with severe periodontitis had significantly higher OR for an increase in the number of MIA components after adjusting confounders (Supplementary Table 2, multivariate model, OR: 2.41, 95% CI 1.34–4.35, p = 0.004), while Eichner index was not significantly associated. In addition, the association between malnutrition defined with serum albumin levels and severe periodontitis were associated significantly (Supplementary Table 3, multivariate model, OR: 2.23, 95% CI 1.11–4.46, p = 0.024).

## Discussion

MIA syndrome is a major fatal factor in hemodialysis patients. However, its association with oral health has not been investigated to date. In this study, severe periodontitis was significantly associated with an increase in MIA components with OR of 2.64 even after adjusting confounding factors. In particular, severe periodontitis was significantly associated with malnutrition and inflammation. Although the association between GNRI, shown as a continuous variable, and the severity of periodontitis was not significant, a significant association was found between severe periodontitis and malnutrition when GNRI was used as a categorical variable and adjusted for confounding factors. Conversely, occlusal support was not significantly associated with an increase in components of MIA syndrome. To the best of our knowledge, this is the first study to analyze the association between oral health and MIA syndrome, suggesting that periodontitis may be involved independent of occlusal support in MIA syndrome in ESRD patients.

Recently, it has been reported that oral diseases affect the systemic condition of ESRD patients^15,17^. Chen et al. reported from a cross-sectional study of patients undergoing hemodialysis in Taiwan that serum albumin and hsCRP levels were significantly associated with periodontitis^18^, and the results of the presented study were consistent with those of this study. Reportedly, mortality from pneumonia^30^, CVD^19^, and all-cause mortality^31^ are significantly higher in hemodialysis patients with periodontitis. The mechanisms have been attributed to periodontal disease-induced bacteremia and increased inflammatory status and risk of aspiration pneumonia due to increased oral bacteria. The present study suggests that periodontitis may exacerbate the vicious spiral caused by MIA syndrome and may be a trigger for the development of immune deficiency or cardiovascular events in patients undergoing hemodialysis.

Inflammation and malnutrition were shown to be closely associated with atherosclerosis and the concept of MIA syndrome was proposed by Stenvinkel et al.^6^. The three components of this syndrome, namely malnutrition, inflammation, and atherosclerosis, significantly and independently affect the prognosis of patients undergoing hemodialysis^32^. Moreover, it is known that the mortality rate increases markedly as the number of each component of the MIA syndrome increases^3,33^. Sueta et al. reported that the OR of patients who have three components for all-cause mortality was 9.65 (95% CI 3.22–28.96, p < 0.001), with reference to those with no components of MIA. In this study, higher prevalence of high inflammation state and malnutrition, but not atherosclerosis, were found in hemodialysis patients with severe periodontitis. This may be because atherosclerotic disease is a cumulative effect of multiple factors. Therefore, the effect of inflammation and malnutrition on atherosclerosis may not be enough detected in this study with the cross-sectional design.

Stenvinkel et al. proposed that two types of malnutrition may occur in patients undergoing hemodialysis^6^. The first type is associated with the uremic syndrome per se or related to factors associated with uremia, such as physical inactivity, underdialysis, dietary restrictions, and psychosocial factors. It is characterized by a modest reduction in serum albumin levels, because of lower protein and energy intake due to uremic toxicity. The second type is the “cytokine-driven” type of malnutrition, which refers to a type of malnutrition due to increased catabolism caused by inflammatory cytokines or increased resting energy expenditure due to comorbidity associated with inflammatory responses^6^. The second type of malnutrition cannot be adequately treated by nutritional therapy alone unless the elimination of concomitant comorbidities and/or cause of chronic inflammation. The hypothesis of this study assumes the possibility of malnutrition due to impaired nutritional intake caused by decreased occlusal support. However, the results of this study indicate that occlusal status is not a significant factor affecting malnutrition in ESRD patients undergoing hemodialysis. In contrast, periodontitis, which is known to increase systemic inflammatory cytokine levels^34^, significantly affected both inflammation and malnutrition status. Therefore, “cytokine-driven” malnutrition might have been the main driver of MIA syndrome in this population.

In patients with MIA syndrome, inflammation leads to malnutrition, and periodontitis may contribute to this pathway in several aspects. First, periodontitis increases oxidative stress^35,36^ and insulin resistance^37^, which may contribute to the burden of systemic inflammation. Second, cytokines produced locally in periodontal tissue by periodontitis and the periodontopathogen bacteria, such as *P. gingivalis*, may migrate into the bloodstream and cause systemic chronic inflammation^38,39^. A systemic chronic inflammatory response decreases in the action of anabolic hormones, such as insulin, and suppression of protein synthesis. Moreover, inflammatory responses not only increase resting energy expenditure, but inflammatory cytokines such as interleukins 1 and 6 also act on the central nervous system, leading to anorexia^40^.

MIA syndrome is a major risk for mortality; however, a fundamental solution has not been established. Although the goal of treatment for patients with MIA syndrome is to control the inflammation, it is often practically difficult to improve the comorbid conditions. In this study, we demonstrated that periodontitis may be an inflammatory burden in patients with MIA syndrome. Periodontitis can be controlled with appropriate periodontal therapy, thereby downregulating systemic CRP levels^41^. The findings of this study suggested that periodontal therapy may have a potential in establishing the treatment strategy for MIA syndrome.

This study has several limitations. First, the occlusal status was based on information from the number and position of the remaining teeth. It did not consider the use of dentures or actual occlusal contact and might not have been able to accurately evaluate the effect of occlusal status. Further study including examination of the actual occlusal status in the oral cavity and quantitative measurement of masticatory function is required. Second, there may be unadjusted confounding factors. Although we adjusted as much as possible for factors assumed to influence MIA syndrome from previous studies, there may be residual confounding factors, such as access to dental care and socioeconomic factors that were not measured in this study.　In addition, Kt/V is an indicator of hemodialysis adequacy and is known to influence mortality^42^ and nutrition status^43^, however, the data of Kt/V were not available for this study and could not be included in the analysis. Third, all participants in the present study were Japanese hemodialysis patients from one clinic. Thus, careful interpretation is needed to apply the presented association between MIA syndrome and periodontitis to the other populations. Forth, the definition of atherosclerosis included a history of CVD events regardless of whether they occurred before or after the start of hemodialysis in this study. This was because we considered that malnutrition, inflammation, and atherosclerosis are conditions that do not start at the time of dialysis introduction but continue from the time of chronic renal failure not yet on dialysis. This should be taken into account when interpreting the results. Finally, the causality cannot be established due to the cross-sectional nature of this study. To elucidate the causality, further cohort or interventional study will be needed.

## Conclusion

This study showed that periodontitis is associated with MIA syndrome in patients undergoing hemodialysis independent of occlusal support, particularly in relation to inflammation and malnutrition. Further cohort and intervention studies are needed to examine a detailed relationship between periodontitis and MIA syndrome in patients undergoing hemodialysis.

## Supplementary Information

Supplementary Tables.

## Data Availability

The datasets analyzed during the current study are available from the corresponding author on reasonable request.
