# Supplementary material for: Malnutrition- inflammation- atherosclerosis (MIA) syndrome associates with periodontitis in end-stage renal disease patients undergoing hemodialysis: a cross-sectional study
Source: Sci Rep. 2023 Jul 21;13:11805. doi: 10.1038/s41598-023-38959-0 (PMC10361958; doi:10.1038/s41598-023-38959-0)
Supplement: Supplementary file 1 — Supplementary Tables. [file 41598_2023_38959_MOESM1_ESM.docx]

| Supplementary Table 1 Clinical case definition used in this study* | | | |
| --- | --- | --- | --- |
| Disease Category | Clinical Definition | | |
|  | CAL |  | PPD |
| Severe periodontitis | ≥ 2 interproximal sites with CAL ≥ 6 mm (not on same tooth) | and | ≥ 1 interproximal sites with PPD ≥ 6 mm |
| Moderate periodontitis | ≥ 2 interproximal sites with CAL ≥ 4 mm (not on same tooth) | or | ≥ 2 interproximal sites with PPD ≥ 5 mm (not on same tooth) |
| Mild periodontitis | ≥ 2 interproximal sites with CAL ≥ 3 mm (not on same tooth) | or | ≥ 2 interproximal sites with PPD ≥ 4 mm (not on same tooth) |
| Healthy | Other than "severe", "moderate" and "mild" |  |  |
| CAL, clinical attachment level; PPD, probing pocket depth | |  |  |
| *Modified with American Academy of Periodontology Centers for Disease Control case definition (Page *et al.* 2007) | | | |

| Supplementary table 2. Ordered logistic regression analysis of the factors influencing the number of components of MIA syndrome including malnutrition categorized with serum albumin levels (N=254) | | | | | | | | |
| --- | --- | --- | --- | --- | --- | --- | --- | --- |
|  |  | Univariate model | | |  | Multivariate model* | | |
|  |  | OR | 95% CI | p-value |  | OR | 95% CI | p-value |
| Periodontitis | Healthy to moderate | Ref. | | |  | Ref. | | |
|  | Severe | 2.38 | 1.35, 4.21 | 0.003 |  | 2.41 | 1.34, 4.35 | 0.004 |
| Eichner index | A | Ref. | | |  | Ref. | | |
|  | B | 1.38 | 0.80, 2.38 | 0.25 |  | 0.87 | 0.48, 1.56 | 0.63 |
|  | C | 2.11 | 1.16, 3.85 | 0.014 |  | 1.26 | 0.62, 2.53 | 0.52 |
| Age (yrs) |  | 1.04 | 1.02, 1.06 | <0.001 |  | 1.03 | 1.01, 1.05 | 0.007 |
| Female |  | 0.73 | 0.46, 1.18 | 0.20 |  | 0.76 | 0.46, 1.26 | 0.29 |
| Smoking Status | Never and former | Ref. | | |  | Ref. | | |
|  | Current | 0.76 | 0.42, 1.36 | 0.35 |  | 0.83 | 0.45, 1.56 | 0.57 |
| BMI | ≥18.5 | Ref. | | |  | Ref. | | |
|  | <18.5 | 1.43 | 0.72, 2.85 | 0.31 |  | 1.89 | 0.93, 3.86 | 0.078 |
| Diabetes |  | 2.15 | 1.36, 3.40 | 0.001 |  | 2.11 | 1.28, 3.50 | 0.004 |
| Hemodialysis vintage (yrs) | | 1.00 | 0.97, 1.04 | 0.87 |  | 1.01 | 0.97, 1.05 | 0.57 |
| Hemoglobin (g/dL) | | 0.98 | 0.88, 1.09 | 0.75 |  | 0.97 | 0.87, 1.08 | 0.57 |
| OR, odds ratio; CI, confidence interval; BMI, body mass index; HD, hemodialysis | | | | | | | | |
| In this model, the number of MIA component(s) (0, 1, 2, and 3) is set as the ordinal dependent variable. | | | | | | | | |
| *Adjusted for all listed variables | | | | | | | | |

| Supplementary table 3. The association between oral health status and malnutrition defined with serum albumin levels (N=254). | | | | | | | | |
| --- | --- | --- | --- | --- | --- | --- | --- | --- |
|  |  | Univariate model | | |  | Multivariate model* | | |
|  |  | OR | 95% CI | p-value |  | OR | 95% CI | p-value |
| Periodontitis | Healthy to moderate | Ref. | | |  | Ref. | | |
|  | Severe | 2.01 | 1.05, 3.85 | 0.034 |  | 2.23 | 1.11, 4.46 | 0.024 |
| Eichner index | A | Ref. | | |  | Ref. | | |
|  | B | 1.15 | 0.63, 2.10 | 0.64 |  | 0.68 | 0.35, 1.34 | 0.27 |
|  | C | 2.25 | 1.16, 4.38 | 0.017 |  | 1.20 | 0.54, 2.67 | 0.65 |
| Malnutrition: serum albumin level <3.5 (g/dL) Malnutrition: serum albumin level <3.5 (g/dL)  * Adjusted for age, sex, smoking status, BMI, diabetes, hemodialysis vintage, hemoglobin | | | | | | | | |
